# An evaluation of LLIN physical integrity and population attitudes towards net use, care and handling during the Magude project in southern Mozambique

**DOI:** 10.1186/s12936-024-04910-5

**Published:** 2024-03-27

**Authors:** Celso Alafo, Lucia Fernandez Montoya, Helena Martí-Soler, Mara Máquina, Arlindo Malheia, Charfudin Sacoor, Ana Paula Abílio, Dulcisaria Marrenjo, Nelson Cuamba, Beatriz Galatas, Pedro Aide, Francisco Saúte, Krijn P. Paaijmans

**Affiliations:** 1 Centro de Investigação em Saúde de Manhiça (CISM), Fundação Manhiça, Maputo, Mozambique; 2 ISGlobal, Barcelona, Spain; 3 Instituto Nacional da Saúde, Ministério da Saúde, Maputo, Mozambique; 4 Programa Nacional de Controlo da Malária, Ministério da Saúde, Maputo, Mozambique; 5 PMI VectorLink Project, Abt Associates Inc., Maputo, Mozambique; 6 Center for Evolution and Medicine, School of Life Sciences, Arizona State University, Tempe, AZ USA; 7 Simon A. Levin Mathematical, Computational and Modeling Sciences Center, Arizona State University, Tempe, AZ USA; 8 Present Address: Global Malaria Program, World Health Organization, Geneva, Switzerland

## Abstract

**Background:**

The Magude Project assessed the feasibility of eliminating malaria in Magude district, a low transmission setting in southern Mozambique, using a package of interventions, including long-lasting insecticidal nets (LLINs). As the efficacy of LLINs depends in part on their physical integrity, this metric was quantified for Olyset® Nets post mass-distribution, in addition to net use, care and handling practices and other risk factors associated with net physical integrity.

**Methods:**

Nets were collected during a cross-sectional net evaluation, nine months after the Magude project commenced, which was 2 years after the nets were distributed by the National Malaria Control Programme (NMCP). The physical integrity of the nets was assessed by counting and sizing the holes at different positions on each net. A structured questionnaire was administered to assess how the selected net was used and treated (care, wash and repair). Net bio-efficacy was assessed following the standard World Health Organization (WHO) cone bioassay procedures.

**Results:**

Out of the 170 Olyset® Nets included in the analysis, 63.5% had been used the night before. The main reason for not using a net was the notion that there were no mosquitoes present. The average number of people using each net was 1.79. Two thirds of the nets had only been washed once or twice since distribution. Most nets (80.9%) were holed and 18% were torn, but none of the risk factors were significantly associated with net integrity, except for presence of mice in the household. Less than half of the participants noticed holes in holed nets, and of those only 38.6% attempted to repair those. None of the six nets that were tested for bio-efficacy passed the WHO threshold of 80% mosquito mortality.

**Conclusion:**

Overall the majority of Olyset® Nets were in serviceable condition two years post-distribution, but their insecticidal effect may have been lost. This study—together with previous evidence on suboptimal access to and use of LLINs in Magude district—highlights that LLINs as an intervention could have been optimized during the Magude project to achieve maximum intervention impact.

**Supplementary Information:**

The online version contains supplementary material available at 10.1186/s12936-024-04910-5.

## Background

Mozambique is one of the four countries that accounted for approximately half of all malaria cases globally in 2021 [1]. It is working collaboratively with South Africa and Eswatini to (a) move South Africa and Eswatini to elimination, and (b) southern Mozambique to pre-elimination. MOSASWA (Mozambique, South Africa and Swaziland, now Eswatini) supports these goals at sub-regional and in-country transmission areas, with the main interventions at the provincial level including prompt diagnosis and effective treatment of confirmed malaria cases, and mosquito vector control by indoor residual spraying (IRS) [2, 3], on top of long-lasting insecticidal nets (LLINs) that are distributed by the country.

Between 2015 and 2018, the Magude project was piloted (in Magude district, southern Mozambique) to assess if local malaria transmission can be interrupted by using all the aforementioned interventions, in addition to mass drug administration (MDA) [3]. Local malaria transmission decreased but was not interrupted [3, 4], which can be attributed to a range of issues, including sub-optimal MDA acceptability [5], the timing and pace of spraying during IRS campaigns [6], insecticide resistance in mosquito vectors, as well as both vector and human behaviors that reduce mosquito exposure to vector control interventions [7–9].

Whereas the project implemented IRS, LLINs were distributed through district-wide mass campaigns by the National Malaria Control Programme (NMCP) in May 2014 and December 2017. Their effective protection in the field depends first on their use, which in turns depends on a population having access to sleeping under an LLIN [10], and both indicators were sub-optimal in Magude district during the project [11]. Two other important factors that contribute to an LLIN’s effectiveness are their ability to (1) prevent vectors from biting humans (providing a physical barrier, disabling or repelling mosquitoes that try to approach the net) and (2) kill mosquitoes (when they come into contact with the insecticides on the net). Holed nets in combination with reduced insecticide availability on net’s surface can increase mosquito blood feeding success [12, 13], which in turn increases the odds of malaria infection [14].

As ample evidence from the field shows that LLIN physical integrity and residual bio-efficacy can decrease rapidly over time after net distribution [15–17], this study aimed to evaluate those LLIN metrics in Magude district 2 years after the nets were distributed (and nine months after the Magude project started). Community net use, care and handling practices and the risk factors explaining the observed net integrity were evaluated as well.

## Methods

### Study site

The study was conducted in Magude district, southern Mozambique, which had a population size of 48,448 people in 2015 [18]. Malaria transmission is seasonal, with a high transmission season lasting from November to April and a low transmission season expanding from May to October. Detailed demographic, health and malaria incidence information has been published elsewhere [18].

During the 2014 LLIN mass distribution campaign (in May), 35,432 LLINs (Olyset Net, Sumitomo Chemical Ltd, Japan; Permanet 2.0, Vestergaard Frandsen, Switzerland) were distributed in the district [18]. An unknown number of LLINs were continuously distributed through the Expanded Programme of Immunization (EPI) and antenatal care services (ANC).

During the Magude project population census in 2015, the majority of the 24,302 nets for which information could be collected (97% of all nets in the district) were Olyset® Nets (77.2%), followed by Permanet® 2.0 (21.1%), Netprotect® (0.5%), Interceptor® (0.1%), Duranet® (0.1%) and DawaPlus® (0.1%), and of 0.9% the brand was unknown. As a previous study conducted in Nampula Province, Mozambique, showed that Olyset® Nets lost their integrity much faster than Permanet® 2.0 [19], the physical integrity of Olyset® Nets that were distributed in Magude district during the 2014 mass-distribution campaign was assessed.

### Cross-sectional evaluation of Olyset® Nets

A cross sectional evaluation of the nets was conducted between May and June 2016, approx. 2 years after their distribution. Data on net physical integrity, community attitudes and practices towards net use, care and repair were collected. The sample size was calculated to detect the percentage of holed nets with 5% confidence, considering an expected percentage of 80% [19] and adjusting for the finite population of 18,748 Olyset® Nets that were recorded during the 2015 population census. This yielded a sample size of 243 LLINs. A 10% margin was added to account for households that may reject participation, absent home owners, or erroneous registration of Olyset® Nets during the population census. This increased the sample size to 267 LLINs. Nets were collected from randomly selected households, where at least one Olyset® Net was identified during the census of the population (8696 households; 82% of all households registered).

During household visits, one adolescent or adult household member (≥ 15 years of age) was asked about the number of nets they received during the mass distribution campaign and that had been used to sleep at least once. Of these, one Olyset® net was selected randomly by assigning a number to each LLIN, writing numbers down on pieces of paper (one number per paper) and asking the interviewee to draw one paper. The selected bednet was taken away for evaluation and a new World Health Organization (WHO)-approved LLIN was given to the household as replacement. All nets were folded and stored individually in plastic bags in an air-conditioned warehouse until their physical integrity assessment.

A structured questionnaire, developed by combining two existing questionnaires [20, 21] (Additional file 1: Table S1), was administered to assess how the selected net was used and treated (care, wash and repair). Questionnaire data (interviews) were collected using tablets and ODK forms and sent daily to a Server Data Base at CISM (Manhiça Health Research Center, Manhiça, Mozambique).

The physical integrity of the collected nets was assessed 7–8 months after their collection (January 2017) by counting and sizing the holes at different positions on the net (roof, left/right long sides, left/right short sides) using PMI’s measurement template [21]. Holes were categorized to one of the following hole groups (in diameter): (1) 0.5–2 cm, (2) 2–10 cm, (3) 10–25 cm, and (4) > 25 cm [20].

### LLIN bio-efficacy testing

The bio-efficacy of 6 randomly selected nets was evaluated in November 2016 (i.e. 5–6 months after net collection; 29–32 months after net distribution), following standard WHO cone bioassay procedures [20] with 2–5 day old unfed *Anopheles arabiensis* females (susceptible KGB strain, maintained at the Instituto Nacional da Saúde, Maputo, Mozambique). Bioassays were conducted at 27 ± 2 °C and 75 ± 10% relative humidity. A total of 8 replicates (cones, with five mosquitoes each) were tested for each net. At least one full bioassay with a control untreated net was run in the same room as the other bioassays during each morning/afternoon of testing treated nets. Mosquito knock-down was recorded 60 min and mortality 24 h post-exposure.

### Data analysis

To ensure that only Olyset® Nets that were distributed during the mass distribution campaign were evaluated, only nets that (a) had the logo of the NMCP (‘PNCM’ in Portuguese) on the label, (b) had the acronym (‘MAG’) written on them with a permanent marker (both are identifiers used by NMCP to identify government-distributed nets), (c) were received for free and (d) did not come from antenatal care services, were include in the analysis.

### Olyset® Net integrity

The ‘proportionate hole index’ (pHI) for LLINs are calculated using WHO guidelines [22]: 1 × (no. of size-1 holes) + (23 × no. of size-2 holes) + (196 × no. of size-3 holes) + (578 × no. of size-4 holes). LLINs were then divided into three categories based on the pHI [23]:

1. good condition (pHI 0–64): no reduction of efficacy compared to an undamaged net;
2. acceptable condition (pHI 65–642): effectiveness somewhat reduced but still provides significantly more protection than no net at all;
3. torn (pHI 643+): protective efficacy for the user is in serious doubt and the net should be replaced as soon as possible.

The mean and median pHI, the proportion of nets that had holes and the proportion of nets in each physical integrity category was calculated. The number of LLINs in good and acceptable condition are combined to estimate the percentage of LLIN in serviceable condition [23]. Number of holes per size category for each net tested are provided in Additional file 2.

### Attitudes towards Olyset® Net use, care and repair

Several questions regarding net use, care and repair practices were asked. For all questions, the frequency of selection of each answer option is provided.

### Risk factors associated with Olyset® Net physical integrity

Factors related to ITN handle, use and care were correlated with ITN categorical physical condition of nets (using Chi-square test) and with net pHI index (using Kruskal–Wallis Test). The Bonferroni and the Holm corrections were applied to correct for multiple bivariate comparisons. The full list of factors evaluated is provided in Additional file 1: Table S2.

### Olyset® Net bio-efficacy

For each net, the mean mosquito mortality across all replicates was calculated. If mortality in the control treatment was (i) between 0 and 10%, the mean mortality was corrected with Abbott’s formula, (ii) > 10%, the test was discarded. Net bio-efficacy data for each net tested are provided in Additional file 3.

## Results

Of the 252 households that were visited, 245 had at least one Olyset® Net that had been used to sleep. Of those, 170 nets were included in the analysis, as 65 nets could not be identified as a campaign net (i.e. label with PNMC logo was missing, the text written with the marker was not visible, or the net was paid for), 7 nets could not be traced back to a household, and 3 nets were Permanet® 2.0 LLINs. This final sample size resulted in slightly higher confidence intervals in our points estimated than expected. Results below are reported with their corresponding confidence intervals or sample sizes to reflect uncertainty.

### Olyset® Net integrity

Overall, 80% (136) of the nets had at least one hole, the remaining 20% (n = 34) had zero holes (or holes that are less than 0.5 cm in diameter, which are not recorded). The median number of holes was 8 (interquartile range; IQR 2–24). The median proportionate hole index (pHI) was 51.5 (IQR 3–289) and the mean pHI 961.8 (standard deviation, SD 8823). Only 55.3% (47.5–62.8) of the nets were classified as ‘good’, 27.1% (20.7–34.5) as ‘acceptable’, and 17.6% (12.4–24.4) as ‘torn’ (i.e. in need of replacement). This means that 82.4% (75.6–87.6) of LLINs were in ‘serviceable’ condition (‘good’ and ‘acceptable’ nets combined).

Interestingly, 65.3% (n = 111) of the participants claimed not seeing holes in their nets, whereas 33.5% (n = 57) did observe holes in their net, and 1.2% (n = 2) did not know if their net had holes. Only 33.5% (n = 57) of the owners of an LLIN with at least one hole, actually saw holes in their net. The percentage of participants whom spotted holes in torn nets was 83.3% (n = 25) while that of participants that spotted holes in damaged nets was 45.6%. The most frequently self-reported cause for holes (Table 1) was mice (29.8%, n = 17), followed by ‘torn by a nail or spike’ (26.3%, n = 15), and that it was ‘pulled and then tore’ (19.3%, n = 11).

**Table 1:** Answers given to the questions that relate to net use, handle and care during the Olyset® Net cross-sectional evaluation in Magude district

Question	Answer options	N	%
Where was the net found, inside or outside the house?	Inside	159	93.5
	Outside	11	6.5
How was the net found?	Lose above the sleeping space	70	41.2
	Hanging and tight with a knot	20	11.8
	Hanging but folded	44	25.9
	Visible but not hanging	19	11.2
	Stored away from the sleeping space	17	10.0
What is the main material to sleep on?	Wooden board (well finished)	2	1.2
	Wooden board (made of sticks)	0	0.0
	Iron surface (metal)	10	5.9
	Foam mattress	105	61.8
	Mat	36	21.2
	Grass	0	0.0
	Floor	6	3.5
	Doesn’t have a fix surface	11	6.5
Was the net used the night before?	Yes	108	63.5
	No	61	35.9
	I don’t know	1	0.6
If not, why not? (n = 61)	There were no mosquitoes	37	60.7
	There was no malaria	0	0.0
	It was hot	2	3.3
	The net was too old or torn	3	4.9
	The net is too dirty	3	4.9
	The net was being washed	1	1.6
	I don’t know	5	8.2
	Other reason	10	16.4
When was the last time that you used the net? (n = 61)	This week	12	19.7
	Last week	13	21.3
	A month ago	15	24.6
	Three months ago	11	18.0
	More than 6 months ago	4	6.6
	More than 1 year ago	4	6.6
	I don’t know	2	3.3
Last week, how often did you use the net? (n = 25)	Every night	14	56.0
	Most nights (5–6 nights)	4	16.0
	Some nights (1–4 nights)	6	24.0
	Never	0	0.0
	I don’t remember	1	4.0
During which period of the year did you use the net?	All year round	134	78.8
	Only with the rainy season	20	11.8
	Only in the dry season	11	6.5
	I don’t know	5	2.9
How often did you use the net during the last summer?	Always	135	79.4
	Most weeks	25	14.7
	Some weeks	10	5.9
	Never	0	0.0
Did you use the net less frequently this summer/rainy season compared to the last summer/rainy season ?	Yes	19	11.2
	No	139	81.8
	I don’t know	12	7.1
If yes, why? (n = 19)	There were no mosquitoes in this rainy season	12	63.2
	It was hotter than in other rainy seasons	1	5.3
	There was no malaria	0	0.0
	The net is more damaged than last year	2	10.5
	The net is dirtier than last year	0	0.0
	Others	4	21.1
How many adults (> 15 years old) slept under a net last night?	0	41	24.1
	1	74	43.5
	2	52	30.6
	3	3	1.8
How many kids (5—15 years old) slept under a net last night?	0	124	72.9
	1	30	17.6
	2	13	7.6
	3	2	1.2
	5	1	0.6
How many kids (< 5 years of age) slept under a net last night?	0	125	73.5
	1	40	23.5
	2	5	2.9
Was this net ever used to sleep outside of the household?	Yes	3	1.8
	No	167	98.2
	I don’t know	0	0.0
If yes, where? (n = 3) *Multiple choice answer, not compulsory by mistake*	It was taken to the field	0	0.0
	It was taken to the beach	0	0.0
	It was taken to the forest	0	0.0
	It was taken to a garner	0	0.0
	It was used to sleep in a hotel	0	0.0
	Others	0	0.0
If yes, when? (n = 3)	All year round	0	0.0
	Only in the rainy season	2	66.7
	Only in the dry season	1	33.3
	I don’t know	0	0.0
Do you tuck the edges of the net under the sleeping space well at night?	Yes	160	94.1
	No	8	4.7
	I don’t know	2	1.2
How do you hang the net?	Rope	118	69.4
	Plastic stripes	31	18.2
	Nails	5	2.9
	Metal wires	10	5.9
	Metal frame	1	0.6
	Wooden frame	0	0.0
	Others	5	2.9
Do you use fire or cook, heat, light a fire in the place where the net is located?	Yes	39	22.9
	No	131	77.1
	I don’t know	0	0.0
If yes, what type? (n = 39) *Multiple choice answer*	Fire from wood	1	2.6
	Fire from coal	0	0.0
	Candle	23	59.0
	Fire lamp with glass	14	35.9
	Lamp without protective glass	3	7.7
	Others	0	0.0
Are there animals inside of the house? *Multiple choice answer*	Cats	71	41.8
	Hens	66	38.8
	Ducks	29	17.1
	Mice	53	31.2
	Dogs	29	17.1
	Others	5	2.9
	No animals	35	20.6
In the last 6 months, have you seen mice or signs thereof? (mice stool, ruminations)	Yes	83	48.8
	No	87	51.2
	I don’t know	0	0.0
Was the net ever washed?	Yes	110	64.7
	No	56	32.9
	I don’t know	4	2.4
If yes, how many times? (n = 110)	I don’t know	6	5.5
	Specify quantity	104	94.5
Number of times (n = 104)	1	43	41.3
	2	36	34.6
	3	18	17.3
	4	2	1.9
	5	2	1.9
	7	1	1.0
	More often	2	1.9
In the last wash, what type of product did you use? (n = 110)	Only water	13	11.8
	Local soap bar	20	18.2
	Detergent (OMO or similar)	77	70.0
	A mix of things (soap and detergent)	0	0.0
	Whitener	0	0.0
	I don’t know	0	0.0
For how long did you dip the net in the water? (n = 110)	I did not dip it	21	19.1
	< 1 h	8	7.3
	> 1 h	71	64.5
	I don’t know	10	9.1
Did you rub or beat the net during the last wash? (n = 110)	Yes	76	69.1
	No	34	30.9
	I don’t know	**0**	
Where did you dry it after the last wash? (n = 110)	Outside on the floor	0	0.0
	Outside on a line	100	90.9
	Outside on a bush or fence	9	8.2
	Inside of the house	1	0.9
	I don’t know	0	0.0
Have you seen any holes in the net?	Yes	57	33.5
	No	111	65.3
	I don’t know	2	1.2
What caused the hole? (n = 57) Multiple choice answer	It tore when caught on a spike or a nail	15	26.3
	It was pulled and tore	11	19.3
	Burned with a candle or spark	4	7.0
	Caused by mice	17	29.8
	Caused by other animals		
	Cut by a knife		
	The hole appeared during the drying of the net	3	5.3
	I don’t know	11	19.3
	Others	1	1.8
Did you ever try to repair the holes in the last 6 months? (n = 57)	Yes	22	38.6
	No	35	61.4
	I don’t know	0	0.0
How where they repaired? (n = 22) *Multiple choice answer*	Sewn	2	9.1
	Tied	21	95.5
	Used a patch	0	0.0
	Others	0	0.0
Who repaired them? (n = 22)	A member of the household	22	100.0
	A tailor	0	0.0
	A friend or relative	0	0.0
	A community volunteer	0	0.0
	Others	0	0.0
What is the main reason for the net not to be repaired? (n = 35)	There was no time	10	28.6
	It was not necessary	4	11.4
	I didn’t have the materials to repair it	6	17.1
	I don’t know how to repair it	15	42.9
	The holes were too big to repair them	0	0.0
	It was not possible to repair the holes	0	0.0
	I don’t want to use the net	0	0.0
	Others	0	0.0
What do you do to prevent the net from getting torn or holes from opening? *Multiple choice answer*	Keep away from kids	58	34.1
	Keep it away from animals	45	26.5
	Roll up or tie up when not in use	128	75.3
	To not mix with food	8	4.7
	Keep it away from fire	14	8.2
	Wash it gently	10	5.9
	Wash it only when it is dirty	10	5.9
	Inspect holes in the net regularly	4	2.4
	Repair the small holes immediately	1	0.6
	It is not possible to prevent holes from happening	1	0.6
	I don’t do anything	2	1.2
	Others	1	0.6

### Olyset® Net repair

Out of the 57 participants that noted holes in their net, 38.6% (n = 22) did attempt to repair them over the last six months (one respondent did not know). Only 12.3% (n = 7) of participants with torn nets tried to repair them over the last six months, whereas 26.3% (n = 15) of people with serviceable nets did.

Participants indicated that the main reasons for not repairing their LLIN were not knowing how to repair their LLIN (42.9%), not having the time (28.6%), not having the tools to repair the net (17.1%), or they thought that repair was not needed (11.4%). Most participants (75.3%) tried to prevent holes by rolling or tying up the net up when not in use, or by keeping it away from children (34.1%) and/or from animals (26.5%).

### Olyset® Net use

Out of the 170 nets, 63.5% had been used the night before, 35.9% not, and one participant did not know. Of the 61 that were not used the night before, 60.7% of the participants responded not using the net because there were no mosquitoes. When asking the participants when the net that was not used the night before, was last used, 41.1% indicated it was used during the past two weeks, 46.2% that it was used in the past 1–3 months, and the remainder was used over 3 months ago. When a net was reported to be used this or last week, 56.6% of the participants indicated it was used every night, 16.0% that it was used most nights (5–6 nights), and the remainder was used 1–4 nights. Regarding net use and seasonality, 78.8% of participants said to use the net year-round, and not only during one specific season, but 11.8% and 6.5% indicated to only use the net during the rainy and dry season, respectively.

Most LLINs (44.4%, n = 64) were used by two household members at the same time, followed by one household member (27.1%, n = 39), three household members (22.2%, n = 32), and the remainder were used by more than three household members (6.3%, n = 15). The mean number of people sleeping under a LLIN the night before was 1.79. Only 1.8% (n = 3) of the nets were used to sleep outside. Of the nets that had been used the night before (n = 104), 96.3% were tuck under the sleeping space.

### Olyset® Net handling

Most nets (93.5%) were found indoors, either loose above the sleeping space (41.2%), hanging but folded (25.9%), hanging and tied in a knot (11.8%), visible in the home but not hanging (11.2%) or stored in sight (11.2%). The most common sleeping surfaces associated with the nets were a foam mattress (61.8%) and a straw mat (21.2%), and the most common way to hang the net above the bed was by using rope (69.4%), plastic strips (18.2%) or metal wire (5.9%). Twenty three percent of respondents used a flame near the net, including a candle or gas light.

### Olyset® Net washing

64.7% of the participants reported the LLIN had been washed. The number of previous washes was as follows: once (41.3%), twice (34.6%), or three or more times (24%). Washing occurred predominantly with powdered detergent (70%), a bar of soap (18.2%) or with just water (11.8%). The majority of the washed nets (64.5%) were soaked for more than one hour, washed without soaking (19.1%), or soaked for less than an hour (7.3%) during the last wash. More than half of the LLINs were scrubbed or beaten during the last wash (69.1%). Drying of nets mainly occurred outdoors on a line (90.9%), but some were dried on a bush or fence (8.2%).

### Risk factors for poor net physical integrity

A total of 24 risk factors were evaluated against the categorical physical condition of the nets and the pHI value. Of the risk factors that were evaluated against the categorical physical condition of the nets, the presence of mice in the house over the last 6 months and the way the net was hung had a p-value of 0.05 (χ^2^). The frequency of use and the number of people sleeping under the net showed a p-value of below 0.1 (χ^2^). After applying the Bonferroni and the Holm correction, none of the factors had a p-value below 0.1. Of the risk factors evaluated against the hole index (PhI), the presence of mice in the house over the last 6 months (Kruskal–Wallis, p = 0.002), whether the net was washed (Kruskal–Wallis, p = 0.04) and the presence of dogs (Kruskal–Wallis, p = 0.05) had a p-value less than 0.05. No other variable had a p-value below 0.1. After applying the Bonferroni and the Holm corrections, only the presence of mice in the house over the last 6 months showed a p-value below 0.1 (Kruskal–Wallis, p = 0.055). The complete list of risk factors that were evaluated against the categorial physical condition of the net and against the pHI are provided in Additional file 1: Table S2.

### Net bio-efficacy

Only the bio-efficacy of 6 nets could be tested due to challenges with maintaining the susceptible mosquito line. No net met the WHO requirement of inducing ≥ 80% mosquito mortality 24 h after exposure, or inducing ≥ 95% knock-down 60 min after exposure. The highest observed mosquito knock-down 1 h after exposure was 12.5%, the highest mortality 24 h after exposure was 35%. Control mortality was ≤ 2.5%. Interestingly, two of the tested nets had never been washed.

## Discussion

Olyset® Nets were the most prevalent (77.1%) bed net brand in Magude district during the Magude project. The present study evaluated the physical integrity of Olyset® Nets and net use, care, and repair practices. Nine months after the start of the Magude project, and 2 years after the mass distribution campaign, 81% of Olyset® Nets (which accounted for 77% of all nets in the district the previous year) had at least one hole, and 17.6% were no longer protective (i.e. were classified as ‘torn’). Their integrity likely decreased further before the next mass distribution campaign (1.5 years later, in December 2017), given the fact that residents did not always notice holes, and did not often repair holes.

The percentage of LLINs with at least one hole was lower compared to the results found for Olyset® Nets in Nampula Province (northern Mozambique) 2 years after their distribution in 2008 [19]. This may be because the current study was a cross-sectional survey whereas the study conducted in Nampula was a prospective durability monitoring study [20]. As approx. 30% of LLINs were lost per year in Magude [11], part of the damaged nets—that are most commonly discarded [24]—may have been missed in the calculations, which likely led to an underestimation of the percentage of LLINs in the damaged and torn categories. Nevertheless, and despite this potential bias towards nets in good conditions, the percentage of LLNs with at least one hole was higher compared to that obtained by durability monitoring studies in Tete province (62.8%; MAGNet® LLINs), and in Inhambane and Nampula provinces (46.8% and 56.3%, respectively; Royal Sentry® LLINs) two years after the mass distribution campaign of 2017 [25], and the percentage of unserviceable nets higher than observed in Inhambane and Nampula (6.6% and 8.2%, respectively). This illustrates that net deterioration may be affected by net brand, but also by local cultural practices (i.e. differences in net use, care and repair) and living conditions [26].

The most frequently reported cause of holes in nets by the participants were mice, and the presence of mice in the household over the last 6 months was significantly associated with the PhI (after the Bonferroni and Holm corrections) and with the categorial physical integrity of the nets (before corrections were applied). This suggests that people are aware of the risk that mice pose to their nets. Rodents are a well-known cause of holes in nets, both in Mozambique [19, 25] and other African countries [26, 27], which suggest that NMCPs could consider increasing community awareness about preventing mice from coming indoors (by e.g. storing food in containers or outside the home) and/or the application or distribution of rodenticides in combination with LLINs in order to extend the life span of LLINs. However, the reasons for holes in the nets are self-reported and may therefore suffer from social desirability and recall bias [28]. Furthermore, there now exists a method to identify the causes of holes through visual inspection [29], which was not available at the time of study.

Several other factors relating to net care and handling [30] and the number of users per net [25] have been associated with reduced net integrity, but none of those factors were significantly associated with net physical integrity in this study. This may be due to the fact that not all participants responded to each net care and handling question, which reduced the sample size for correlation analysis.

Detailed investigations are warranted to determine the protective efficacy of LLINs across Mozambique, as studies have shown that malaria incidence levels can be higher among users of holed nets [14, 31], although the scientific evidence is not always in agreement [32]. A better understanding of the effect of holed nets on malaria infection risk is needed to understand the real protection that holed LLINs confer, to ascertain the value of implementing strategies to preserve net integrity until the next mass distribution campaign and to reconsider the frequency of LLIN distribution.

The fact that 66.5% of participants did not notice holes in their holed nets, and that only 38.6% of the participants that noticed holes attempted to repair them, shows the need to raise community awareness of the importance of regularly inspecting nets and repair holes in order to prolong their net’s serviceable life. In contrast, the fact that more than 83.3% of participants did claim seeing holes in nets that needed replacement (torn) and 45.6% in nets that needed repairing (in damaged state) suggests that communities may be able to self-report bednets that are in poor condition. If this fact is confirmed through further studies, the implementation of systems whereby people can report their torn or damaged net and obtain either a new net or get help with repairs (e.g. continuous net distribution, repair points, services for net repair provided by local tailors or community health workers) could help to maintain the good physical integrity of nets in the field after mass distribution and hence preserve the maximum protection LLINs confer.

Regarding LLIN use, most participants reported to use the net year-round, while previous data over multiple points in time showed that net use was lower during the dry season [7, 11]. Nevertheless, the findings in this and previous studies in the district highlight that there is room for improving LLIN use, especially during the dry transmission when the interruption of local malaria transmission may be more feasible. Given that the main reported reason for not using the net was that there were no mosquitoes, increasing LLIN use will require strategies to improve the disease risk perception in communities. Finally, an average of 1.8 people per net was reported in the district, which is in line with the recommended method of assessing full universal coverage (1.8 people per net). However, a previous study showed that LLIN access never exceeded 76.3%, and that use varied seasonally between 40 and 76.4% [11].

LLINs are designed to retain their insecticidal activity under 20 standard washes as per WHO protocol [20]. Seventy-five percent of the participants who washed their nets merely washed it once or twice since distribution two years ago, a practice that could preserve the bio-efficacy of nets for 3 years (expected serviceable life of an LLIN). However, participants washed them using detergent powder and dried them under the sun, behaviours that have been found to reduce the bio-efficacy of nets [33]. This suggests that the majority of LLINs will not retain their insecticidal effect for 20 washes in Magude. Although only the bio-efficacy of 6 nets could be tested, the results indicate that the nets had very low bio-efficacy (under 35% 24 h mosquito mortality) by month 29–32 post-distribution, thus failing to meet the WHO 3-year cone bio-efficacy requirement. However, when mosquito mortality in cone bioassays is below 80%, the WHO also recommends conducting tunnel tests [34, 35], which were not performed in this study. As such, the actual field performance of Olyset® Nets may have been underestimated. To further illustrate, a previous study coordinated by WHOPES and the WHO Global Malaria Program in seven countries showed that  while 33% of the tested Olyset® Nets failed to meet the target 80% mortality in cone bioassays, half of those nets did meet the WHO criteria for the tunnel test [35]. In addition, the chemical content of the LLINs was not analysed. This would have allowed for a more accurate interpretation of the bioefficacy results. It is known that the release rate of permethrin from Olyset® Nets is low [36], and that the low observed bio-efficacy in this study may be similar to the efficacy of a new Olyset® Net.

The proportion of torn nets, combined with the number of serviceable nets that were not repaired and the apparent loss of bio-efficacy, can lead to no or smaller reductions in human-vector contact and reduced mosquito mortality inside a given household. This may have favored the predominant vector species, such as *An. arabiensis* [7, 8] as well as the local pyrethroid resistant *Anopheles funestus *sensu stricto and *Anopheles parensis* [37, 38] by allowing them to feed on humans while under the net, thus contributing to sustained malaria transmission in Magude district [12, 13].

The evaluation of the LLIN use, handle, care and repair and risk factor for poor physical integrity is, however, affected by the fact that survey questions were answered by an adolescent or adult member of the household, which may not have been the user of the net under evaluation. In addition, one should not overinterpret the bio-efficacy results, as these are based on a low number of nets tested and the WHO cone bioassay only (and no tunnel tests were conducted). Finally, while Olyset® Nets accounted for 77% of the nets in the district, it would have been useful to collect similar data for the other LLIN brands that were found in the district. This would have allowed for a deeper understanding of the actual protection that LLINs conferred during the Magude project.

## Conclusion

Overall, the majority of Olyset® Nets that were investigated during the Magude project were in serviceable condition two years post-distribution, but their insecticidal effect may have been lost. Improving community awareness after net mass-distribution campaigns about the importance of inspecting for and repairing holes, implementing strategies to support communities in repairing or replacing their torn nets and improving washing and drying practices may improve LLIN efficacy. As such, it would be beneficial for NMCPs to monitor net integrity and bio-efficacy annually after each net distribution campaign, to allow for the implementation of additional strategies to optimize LLIN efficacy during the three years between mass-distribution campaigns. Apart from the aforementioned community awareness campaigns, additional strategies could include the continuous distribution of nets in so-called top-up campaigns (to replace torn nets), or campaigns tailored to specific risk factors that are associated with net integrity, such as raising awareness about the role of mice in net integrity. This study, together with previous evidence of suboptimal access to and use of LLINs in Magude district [11], clearly highlights that there are gaps in protection by LLINs that can be overcome [39], and that LLINs as an intervention could have been optimized during the Magude project to achieve maximum impact.

### Supplementary Information

**Additional file 1: Table S1.** Questionnaire used during the Olyset® cross-sectional evaluation (original questionnaire in Portuguese). **Table S2.** Factors included in the net physical integrity risk factor analysis.

**Additional file 2.** Data file with number of holes per size category for each net tested.

**Additional file 3.** Data file with net bio-efficacy data for each net tested.

## Data Availability

The data supporting the conclusions of this manuscript are available in the table in the main text and the three additional files (S1-S3).
