# Supplementary material for: An evaluation of LLIN physical integrity and population attitudes towards net use, care and handling during the Magude project in southern Mozambique
Source: Malar J. 2024 Mar 27;23:87. doi: 10.1186/s12936-024-04910-5 (PMC10967156; doi:10.1186/s12936-024-04910-5)
Supplement: Supplementary file 1 — Additional file 1: Table S1. Questionnaire used during the Olyset® cross-sectional evaluation (original questionnaire in Portuguese). Table S2. Factors included in the net physical integrity risk factor analysis. [file 12936_2024_4910_MOESM1_ESM.docx]

**Table S1.** Questionnaire used during the Olyset® cross-sectional evaluation (original questionnaire in Portuguese)

| **#** | **Variable / Field Name** | **Field Label**  Field Note | **Field Attributes (Field Type, Validation, Choices, Calculations, etc.)** |
| --- | --- | --- | --- |
| 1 | uri | Record ID | text |
| 2 | home_visited | Check whether the house that you are visiting is in the list | radio   \| 1 \| Yes \| \| --- \| --- \| \| 2 \| No \| |
| 3 | local_geografico_lng | Geographical coordinates: longitud | text |
| 4 | local_geografico_alt | Geographical coordinates: altitude | text |
| 5 | local_geografico_acc | Geographical coordinates: accuracy of location | text |
| 6 | local_geografico_lat | Geographical coordinates: latitude | text |
| 7 | data_inquerito | 2. Date | text (date_dmy) |
| 8 | codigo_inquiridor | 3. Code of interviewer | text (integer, Min: 0, Max: 999) |
| 9 | num_agregado | 4.House number | text |
| 10 | select_quem_resp | 5.Who is responding to the questions? | radio   \| 1 \| Household chief \| \| --- \| --- \| \| 2 \| Net user \| \| 3 \| Guardian/legal tutor of the net user \| \| 4 \| Another adult from the household \| |
| 11 | nome_agregado | 6.Name of the household chief | text |
| 12 | adultos15 | adults> 15 years of age | text |
| 13 | anos_5_15 | 5-15 years of age | text |
| 14 | menor_5 | < 5 years | text |
| 15 | nr_rede_agr_num | 8. How many nets does this household have? | text (integer, Max: 50) |
| 16 | quant_redes_pncm_2014 | 9.How many nets did the household receive from the NMCP on the 2014 mass distribution campaign? | text (integer) |
| 17 | quant_redes_pncm | 10.How many of the nets received during the 2014 campaign continue to be present in the household? | text (integer) |
| 18 | quant_redes_dorm | 11.From the nets distributed in the 2014 campaign and that continue to be present in the household, how many are being used to sleep? | text (integer) |
| 19 | log_pncm | 1.Does the net have the NMCP logo? | radio   \| 1 \| Yes \| \| --- \| --- \| \| 2 \| No \| |
| 20 | mark_visivible | 2.Is the marker sign still visible? | radio   \| 1 \| Yes \| \| --- \| --- \| \| 2 \| No \| \| 3 \| No, but it had it in the past \| |
| 21 | receive_2014 | 3. Was this net received through the 2014 mass distribution campaign? | radio   \| 1 \| Yes \| \| --- \| --- \| \| 2 \| No \| \| 3 \| I don’t know \| |
| 22 | net_free | 4. Did you receive the net for free? | radio   \| 1 \| Yes \| \| --- \| --- \| \| 2 \| No \| \| 3 \| I don’t know \| |
| 23 | net_serv_neonatais | 5. The net was acquired during the first antenatal care visit? | radio   \| 1 \| Yes \| \| --- \| --- \| \| 2 \| No \| \| 3 \| I don’t know \| |
| 24 | sleep_used_net | 6. Was this net ever used? | radio   \| 1 \| Yes \| \| --- \| --- \| \| 2 \| No \| \| 3 \| I don’t know \| |
| 25 | image_1 | 1.Take a picture of the selected net as it was found | descriptive |
| 26 | in_out | 2. Where was the net found, inside or outside the house? | radio   \| 1 \| Inside \| \| --- \| --- \| \| 2 \| Outside \| |
| 27 | how_found | 3. How was the net found? | radio   \| 1 \| Lose above the sleeping space \| \| --- \| --- \| \| 2 \| Hanging and tight with a knot \| \| 3 \| Hanging but folded \| \| 4 \| Visible but not hanging \| \| 5 \| Stored away from the sleeping space \| |
| 28 | ground_type_sleep | 4. What is the your main sleeping place and material of it?? | radio   \| 1 \| Wooden board (well finished); \| \| --- \| --- \| \| 2 \| Wooden board (made of sticks) \| \| 3 \| Iron surface (metal) \| \| 4 \| Foam mattress \| \| 5 \| Mat \| \| 6 \| Grass \| \| 7 \| Floor \| \| 8 \| Doesn’t have a fix surface \| |
| 29 | last_night_used | 5. Was the net used the night before? | radio   \| 1 \| Yes \| \| --- \| --- \| \| 2 \| No \| \| 3 \| I don’t know \| |
| 30 | why_night | 6.If not, why not? | radio   \| 1 \| There was no mosquitoes \| \| --- \| --- \| \| 2 \| There is no malaria now \| \| 3 \| It was hot \| \| 4 \| The net was too old or torn \| \| 5 \| The net is too dirty \| \| 6 \| The net was being washed \| \| 7 \| I don’t know \| \| 8 \| Other reason, specify \| |
| 31 | other_why_night | Specify | text |
| 32 | last_uused | 6.1. When was the last time that you used the net? | radio   \| 1 \| This week \| \| --- \| --- \| \| 2 \| Last week \| \| 3 \| A month ago \| \| 4 \| Three months ago \| \| 5 \| More than 6 months ago \| \| 6 \| More than 1 year ago \| \| 7 \| I don’t know \| |
| 33 | freq_use_net | 7. Last week, how often did you use the net? | radio   \| 1 \| Every night \| \| --- \| --- \| \| 2 \| Most nights (5-6 nights); \| \| 3 \| Some nights (1-4 nights) \| \| 4 \| Never (0 nights) \| \| 5 \| I don’t remember \| |
| 34 | year_per_usenet | 8. During which period of the year did you use the net? | radio   \| 1 \| All year round \| \| --- \| --- \| \| 2 \| Only with the rainy season \| \| 3 \| Only in the dry season \| \| 4 \| I don’t know \| |
| 35 | freq_use_net_summer | 9. How often did you use the net during the last summer? | radio   \| 1 \| Always; \| \| --- \| --- \| \| 2 \| Most weeks \| \| 3 \| Some weeks \| \| 4 \| Never \| |
| 36 | sum_rain | 10. Did you use the net less frequently this summer/rainy season compared to the last summer/rainy season ? | radio   \| 1 \| Yes \| \| --- \| --- \| \| 2 \| No \| \| 3 \| I don’t know \| |
| 37 | if_yes_why | 11. If yes, why? | checkbox   \| 1 \| if_yes_why___1 \| There was no mosquitos in this rainy season \| \| --- \| --- \| --- \| \| 2 \| if_yes_why___2 \| It was hotter than in other rainy seasons \| \| 3 \| if_yes_why___3 \| There was no malaria \| \| 4 \| if_yes_why___4 \| The net is more damaged than last year \| \| 5 \| if_yes_why___5 \| The net is dirtier than last year \| \| 6 \| if_yes_why___6 \| Others, specify \| |
| 38 | other_if_yes_why | 11.1. Specify | text |
| 39 | num_sleep_mr | 12. How many adults (>15 ) slept under aa net last night? | text (integer) |
| 40 | num_sleep_chil_1 | 13.How many kids (5 - 15 years) slept under aa net last night? | text (integer) |
| 41 | num_sleep_chil_2 | 14. How many kids (< 5 years of age) slept under aa net last night? | text (integer) |
| 42 | out_sleepin | 15. Was this net ever used to sleep outside of the household? | radio   \| 1 \| Yes \| \| --- \| --- \| \| 2 \| No \| \| 3 \| I don’t know \| |
| 43 | if_yes_where | 16. If yes, where? | checkbox   \| 1 \| if_yes_where___1 \| It was taken to the field \| \| --- \| --- \| --- \| \| 2 \| if_yes_where___2 \| It was taken to the beach \| \| 3 \| if_yes_where___3 \| It was taken to the forest \| \| 4 \| if_yes_where___4 \| It was taken to a garner \| \| 5 \| if_yes_where___5 \| It was used to sleep in a hotel \| \| 6 \| if_yes_where___6 \| Others, especificy \| |
| 44 | other_if_yes_where | 16.1 | text |
| 45 | if_yes_what_per | 17.If yes, when? | radio   \| 1 \| All year round \| \| --- \| --- \| \| 2 \| Only in the rainy season; \| \| 3 \| Only in the dry season; \| \| 4 \| I don’t know \| |
| 46 | org_border_night | 18. Do you tuck the edges of the net under the sleeping space well at night? | radio   \| 1 \| Yes \| \| --- \| --- \| \| 2 \| No \| \| 3 \| I don’t know \| |
| 47 | how_fix_net | 19. How do you hang the net? | radio   \| 1 \| Rope \| \| --- \| --- \| \| 2 \| Plastic stripes \| \| 3 \| Nails \| \| 4 \| Metal wires \| \| 5 \| Metal frame \| \| 6 \| Wooden frame \| \| 7 \| Others, specify \| |
| 48 | other_how_fix_net | 19.1. Specify | text |
| 49 | fire_near_net | 20. Do you use fire or cook, heat, light a fire in the place where the net is located? | radio   \| 1 \| Yes \| \| --- \| --- \| \| 2 \| No \| \| 3 \| I don’t know. \| |
| 50 | if_yes_fire | 21. If yes, what type? | checkbox   \| 1 \| if_yes_fire___1 \| Fire from wood \| \| --- \| --- \| --- \| \| 2 \| if_yes_fire___2 \| Fire from coal \| \| 3 \| if_yes_fire___3 \| Candle \| \| 4 \| if_yes_fire___4 \| Fire lamp with glass \| \| 5 \| if_yes_fire___5 \| Lamp without protective glass \| \| 6 \| if_yes_fire___6 \| Others, especificy \| |
| 51 | other_if_yes_fire | 21.1. Especificar | text |
| 52 | animals_in | 22 Are there animals inside of the house? *Note: Multiple choice answer.* | checkbox   \| 1 \| animals_in___1 \| Cats \| \| --- \| --- \| --- \| \| 2 \| animals_in___2 \| Hens \| \| 3 \| animals_in___3 \| Ducks \| \| 4 \| animals_in___4 \| Mice \| \| 5 \| animals_in___5 \| Dogs \| \| 6 \| animals_in___6 \| Others \| \| 7 \| animals_in___7 \| No animals \| |
| 53 | other_animals_in | 22.1 Especificy | text |
| 54 | last_6_m_mices_in | 23. In the last 6 months, have you seen mice or signs thereof? (mice stool, ruminations) | radio   \| 1 \| Yes \| \| --- \| --- \| \| 2 \| No \| \| 3 \| I don’t know \| |
| 55 | washed_once | 1. Was the net ever washed? | radio   \| 1 \| Yes \| \| --- \| --- \| \| 2 \| No \| \| 3 \| I don’t know. \| |
| 56 | yes_washed | 2. If yes, how many times? | radio   \| 1 \| I don’t know \| \| --- \| --- \| \| 2 \| Specify quantity \| |
| 57 | num_times_washed | Number of times | text |
| 58 | kind_soap | 3. In the las wash, what type of product did you use? | radio   \| 1 \| Only water \| \| --- \| --- \| \| 2 \| Local soap bar \| \| 3 \| Detergent (OMO or similar) \| \| 4 \| A mix of things (soap and detergent) \| \| 5 \| Whitener \| \| 6 \| I don’t know \| |
| 59 | time_wet | 4. For how long did you dip the net in the water? | radio   \| 1 \| I did not dip it \| \| --- \| --- \| \| 2 \| < 1h \| \| 3 \| >1h \| \| 4 \| I don’t know \| |
| 60 | beated_or_not | 5. Did you rub or knock the net during the last wash? | radio   \| 1 \| Yes \| \| --- \| --- \| \| 2 \| No \| \| 3 \| I don’t know. \| |
| 61 | last_wash_place_dry | 6. Where did you dry it after the last wash? | radio   \| 1 \| Outside on the floor \| \| --- \| --- \| \| 2 \| Outside on a line \| \| 3 \| Outside on a bush or fence \| \| 4 \| Inside of the house \| \| 5 \| I don’t know \| |
| 62 | net_hole | 1. Have you seen any holes in the net? | radio   \| 1 \| Yes \| \| --- \| --- \| \| 2 \| No \| \| 3 \| I don’t know \| |
| 63 | hole_root | 2. What caused the hole?  *Note: Multiple choice answers* | checkbox, Required   \| 1 \| hole_root___1 \| It tore when caught on a spike or a nail; \| \| --- \| --- \| --- \| \| 2 \| hole_root___2 \| It was pulled and tore; \| \| 3 \| hole_root___3 \| Burned with a candle or spark \| \| 4 \| hole_root___4 \| Caused by mice \| \| 5 \| hole_root___5 \| Caused by other animals \| \| 6 \| hole_root___6 \| Cut by a knife \| \| 7 \| hole_root___7 \| The hole appear during the drying of the net \| \| 8 \| hole_root___8 \| I don’t know \| \| 9 \| hole_root___9 \| Others \| |
| 64 | other_hole_root | 2.1. Specify | text |
| 65 | repair_holes_6 | 3. Did you ever try to repair the holes in the last 6 months? | radio   \| 1 \| Yes \| \| --- \| --- \| \| 2 \| No \| \| 3 \| I don’t know \| |
| 66 | how_repaired | 4. How where they repaired?  *Note: Multiple choice answers* | checkbox   \| 1 \| how_repaired___1 \| Sewn \| \| --- \| --- \| --- \| \| 2 \| how_repaired___2 \| Tied \| \| 3 \| how_repaired___3 \| Used a patch \| \| 4 \| how_repaired___4 \| Others \| |
| 67 | other_how_repaired | 4.1. Specify | text |
| 68 | who_reapaired | 5. Who repaired them?  *Note: Multiple choice answers* | checkbox   \| 1 \| who_reapaired___1 \| A member of the household \| \| --- \| --- \| --- \| \| 2 \| who_reapaired___2 \| A tailor \| \| 3 \| who_reapaired___3 \| A friendo r relative \| \| 4 \| who_reapaired___4 \| A community volunteer \| \| 5 \| who_reapaired___5 \| Others \| |
| 69 | other_who_reapaired | 5.1. Specify | text |
| 70 | rootcause_repair | 6. What is the main reason for the net not to be repaired? | radio   \| 1 \| There was no time \| \| --- \| --- \| \| 2 \| It was not necessary \| \| 3 \| I didn’t have the materials to repair it \| \| 4 \| I don’t know how to repair it \| \| 5 \| The holes were too big to repair them \| \| 6 \| It was not possible to repair the holes \| \| 7 \| I don’t want to use the net \| \| 8 \| Others \| |
| 71 | other_rootcause_repair | 6.1. Specify | text |
| 72 | prevent_from_holes | 7. What do you do to prevent the net from getting torn or holes from opening? *Note: Multiple choice answers* | checkbox   \| 1 \| prevent_from_holes___1 \| Keep away from kids; \| \| --- \| --- \| --- \| \| 2 \| prevent_from_holes___2 \| Keep it away from animals \| \| 3 \| prevent_from_holes___3 \| Roll up or tie up when not in use \| \| 4 \| prevent_from_holes___4 \| To not mix with food \| \| 5 \| prevent_from_holes___5 \| Keep it away from fire \| \| 6 \| prevent_from_holes___6 \| Wash it gently; \| \| 7 \| prevent_from_holes___7 \| Wash it only when it is dirty \| \| 8 \| prevent_from_holes___8 \| Inspect holes in the net regularly \| \| 9 \| prevent_from_holes___9 \| Repair the small holes inmediately \| \| 10 \| prevent_from_holes___10 \| It is not possible to prevent holes from happening \| \| 11 \| prevent_from_holes___11 \| I don’t do anything \| \| 12 \| prevent_from_holes___12 \| Others \| |
| 73 | other_prevent_from_holes | 7.1. Specify | text |
| 74 | num_batch_net | Introduce the net’s batch number | text |
| 75 | date_adqr_bnet | Introduce the net of net acquisition | text |
| 76 | fam_cons_inf | What is the number of the inform consent? | text |
| 77 | inq_cons_inf | What is the number of the inform consent that stays with the interviewer | text |
| 78 | questionrio_da_utilizao_das_redes_mosquiteiras_complete | Section Header: *Form Status*  Complete? | dropdown   \| 0 \| Incomplete \| \| --- \| --- \| \| 1 \| Unverified \| \| 2 \| Complete \| |

**Table S2.** Factors included in the net physical integrity risk factor analysis

| **Variable** | **Value options** |
| --- | --- |
| How the net was found | Loose above the sleeping surface, hanging an tight with a knot, hanging but folded, visible but not hanging, in storage |
| Surface used to sleep | Wooden smooth surface, wooden sticks, iron plate, foam mattress, matting, grass, on the floor, there is no fix surface |
| Whether the net was used the night before the interview | Yes, no, I don’t know |
| Last week, how often was the net used? | Every night, almost all nights (5-6 nights), some nights (1-4 nights), we didn’t use it (0 nights), I don’t know |
| Whether the net was used to sleep outdoors | Yes, no, I don’t know |
| Whether the net is tuck under the bed | Yes, no, I don’t know |
| How is the net hanged | Rope, plastic stripes, nails, metal hocks, around metal structure, around wooden structure, others |
| Whether fire is lit up in the place where the net was found | Yes, no, I don’t know |
| Whether mice have been observed in the households in the last 6 months | Yes, no, I don’t know |
| Whether the net was ever washed | Yes, no, I don’t know |
| How many times the net was washed |  |
| Whether the net was beaten | Yes, no, I don’t know |
| Place where the net was left to dry | On the floor, hanging on a line, on a bush or fence, inside of the house, I don’t know |
| Presence of the following animals   - cats - hens - duck - mice - dogs - others - no animals | Yes, no |
